# p.Gly743Val Mutation in COL4A1 Is Responsible for Familial Porencephaly and Severe Hypermetropia

**DOI:** 10.3389/fneur.2020.00827

**Published:** 2020-09-11

**Authors:** Pasquale Scoppettuolo, Noémie Ligot, Vanessa Wermenbol, Patrick Van Bogaert, Gilles Naeije

**Affiliations:** ^1^Neurology Department, ULB-Hôpital Erasme, Université Libre de Bruxelles (ULB), Brussels, Belgium; ^2^Neuropediatrics Department, ULB-Hôpital Erasme, Université Libre de Bruxelles (ULB), Brussels, Belgium; ^3^Pediatry Department– CHU Angers, Angers, France

**Keywords:** COL4A1, Type IV collagen, familial porencephaly, ocular malformations, variable expressivity

## Abstract

COL4A1 is an essential component for basal membrane stability. Exon mutations of the COL4A1 genes are responsible for a broad spectrum of cerebral, ocular, and systemic manifestations. We describe here the phenotype of a likely pathogenic gene variant, p.Gly743Val, which is responsible for a missense mutation in the COL4A1 gene exon 30 in a three generation family with severe hypermetropia and highly penetrant porencephaly in the absence of systemic manifestations. This report highlights both the broad spectrum of COL4A1 mutations and the yield of testing the COL4A1 gene in familial ophthalmological and brain disorders.

## Introduction

COL4A1 is an essential component for basal membrane stability and exon mutations of COL4A1 gene mutations are responsible for a broad spectrum of systemic manifestations characterized by small vessel involvement of variable severity, including neurological (1) [porencephaly (2–4), hemorrhage (2, 5–7) and aneurysms (8)], ophthalmological (9–12) (retinal artery tortuosity, Axenfeld Rieger anomalies, cataracts, and severe hypermetropia), renal (13) (renal cysts, and microscopic hematuria), and systemic (13) findings (cramps with a high creatine kinase level [CK], Raynaud's phenomenon, and arrhythmias). The inheritance pattern is autosomal dominant (14) and age-dependent with almost 100% penetrance. The expressivity of the disease is highly variable with high intra- and inter-familial variability (2). To date, over 50 pathogenic or likely pathogenic variants have been described in the COL4A1 gene, most of them missense (2). Since fewer than 100 families have been reported, the exact prevalence of COL4A1-related disorders is not well-established. Here we report a family in which three siblings presented severe hypermetropia and porencephaly. Probands' father had severe hypermetropia and bilateral cataracts. Molecular analysis in the father disclosed a heterozygous variant c.2228G>T (p.Gly743Val) in exon 30 of the COL4A1 gene that segregated with the phenotype.

## Case Presentation

**Figure 1:**
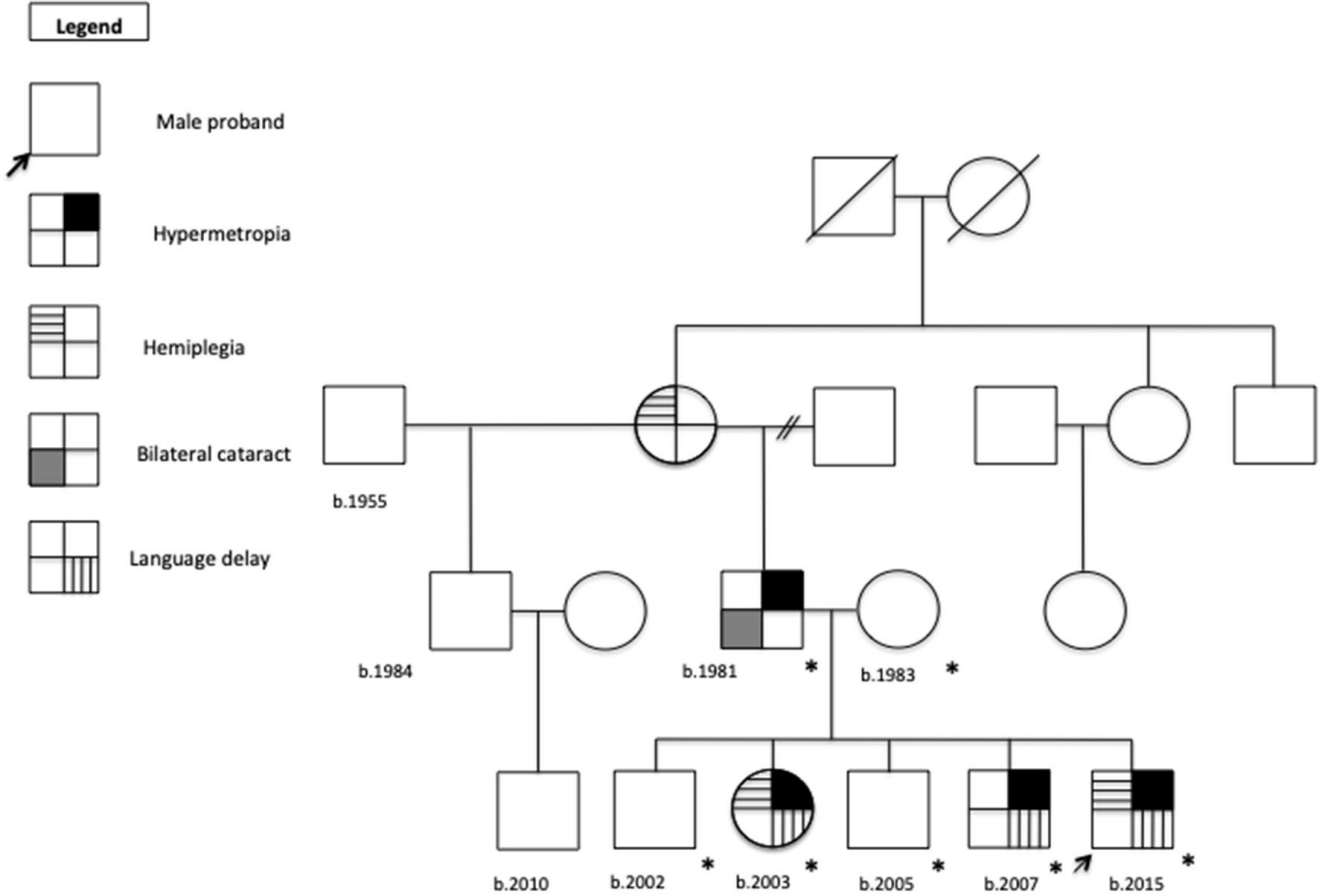
Familial pedigree.

**Figure 2:**
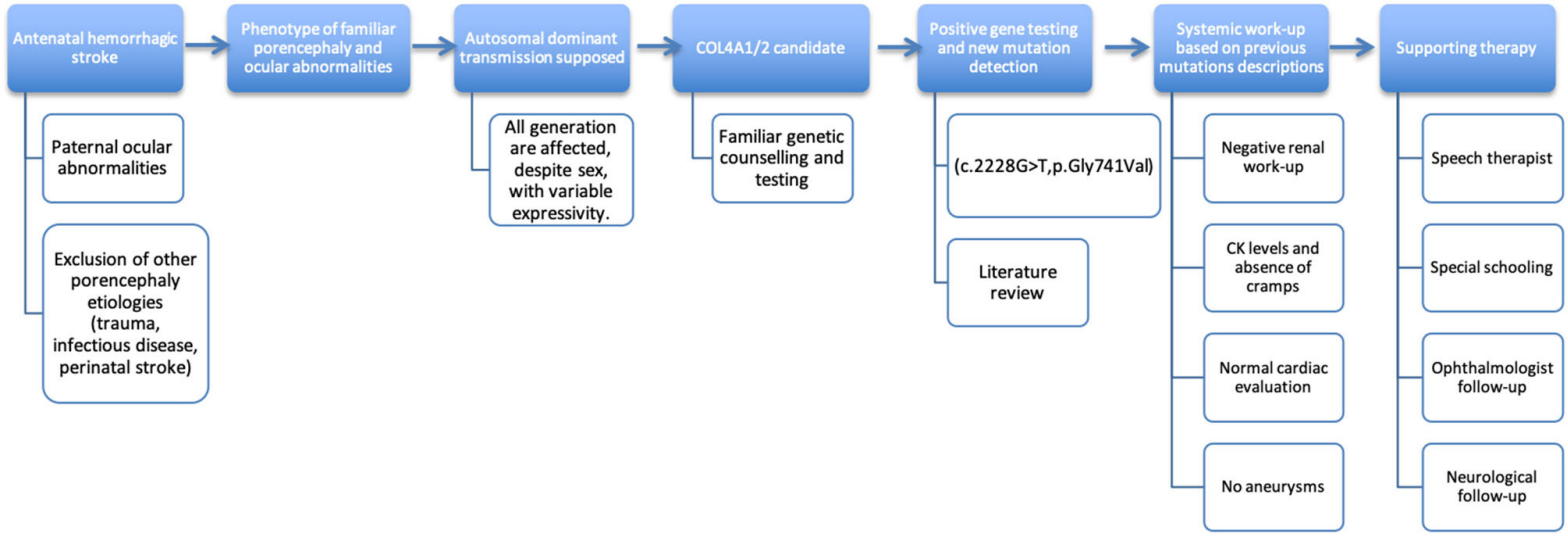
Timeline.

Standardized (15) familiar pedigree is showed in Figure 1. The timeline for the clinical examination and ancillary tests performed is illustrated in Figure 2.

**Figure 3:**
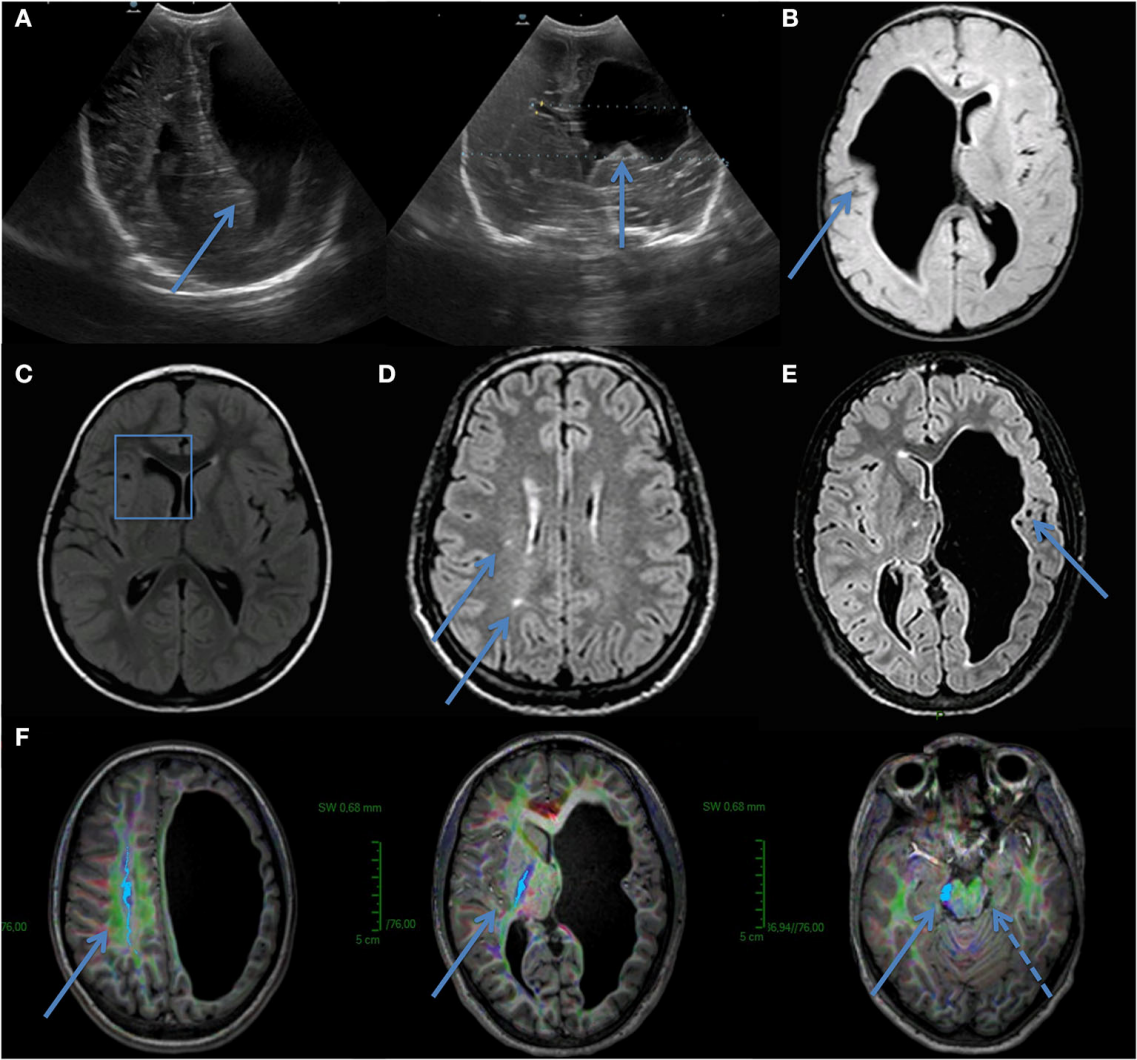
Ultrasound *in utero* from IV-6 **(A)**. Image showed ventricular asymmetry and brain MRI confirmed right frontotemporal dilatation **(B)**. IV-5–Brain MRI revealing porencephalic cyst of frontal horn of lateral right ventricle **(C)**. **(D)** III- 3–Brain MRI showed small asymptomatic lesions in white matter. No microbleeds or cystic cavities were found. **(E,F)** IV-3–Brain MRI showed left frontotemporal dilatation and diffusion tensor imaging (DTI) sequences demonstrated no left corticospinal tract (cranio-caudal fibers, indigo, with arrows). A dashed arrow indicates secondary atrophy in the left cerebral peduncle.

IV-6 was born at 35 weeks after a pregnancy marked by gestational diabetes. He underwent at birth neurosonography for axial hypotonia that revealed ventricular asymmetry and right frontotemporal dilatation (Figure 3). At 1 month of age, a neuropediatric examination disclosed normal neck muscle tonus, normal Moro reflex, bilateral placing reaction, and open hands. Ten months later, the left hemiparesis was observed with a lack of voluntary prehension on his left side without spasticity. At 2 years old, IV-6 presented obvious left hemiparesis but could move without help. Oral expression was reduced and neuropsychological testing revealed language delay with a prominent expression deficit.

III-3 was asymptomatic but for severe hypermetropia and bilateral cataracts.

II-2 had a limp since childhood attributed to forceps delivery.

IV-3 was diagnosed with ventriculomegaly *in utero*. Born at term after a 39-week pregnancy, IV-3 had an unremarkable first clinical evaluation at 3 months. One year later, right hemiparesis became clinically evident with a lack of right voluntary hand prehension in association with right hemineglect. She, then, developed seizures which were controlled by valproic acid. Neuropsychological tests disclosed language delay and learning difficulties requiring speech therapy at the age of 9 years. She also showed severe hypermetropia.

IV-5 had microcephaly without motor deficits, a language delay, a mental retardation (IQ of 62) that required adapted schooling, and severe hypermetropia.

## Diagnostic Assessment

### Brain Magnetic Resonance

Brain magnetic resonance imaging (MRI) scans were carried out on a three Tesla Brain MRI (Achieva, Ingenia; Philips Healthcare, Best, The Netherlands). Berg's criteria was used for porencephaly (16, 17) and white matter hyperintensities were characterized as in Fazekas et al. (18) and Staals et al. (19)

The brain MRI of IV-6 disclosed a large right-sided frontoparietal cavity (Figure 3B) with communication to the lateral ventricle, isosignal to CFS. IV-3 had a left hemisphere porencephalic cyst and the lack of evidence of a left corticospinal tract on tractography (Figures 3E,F), IV-5 had a porencephalic cyst on the right lateral ventricle (Figure 3C), and III-3 had leukoencephalopathy (Figure 3D).

### Ophthalmological Findings

Full ophthalmological evaluations including slit lamp and fundoscopy were realized and disclosed for bilateral hypermetropia in IV-3 [15 dioptre (D)], IV-6 (8.5 D), IV-5 (10 D), and III-3 (7 D).

### Diagnostic Challenges

Other causes of porencephaly were ruled out [maternal alloimmunization, trauma, peri-natal cerebral ischemia (normal Apgar scores at birth), and negative TORCH complex tests].

### Genetic Analysis

The COL4A1 and COL4A2 genes were screened in proband IV-6. Molecular analysis was performed on a gDNA level by means of PCR amplification of all the coding exons and the flanking intron region. Illumina's Sequencing by Synthesis (SBS) technology (MiSeq Personal Sequencer, Illumina) analyzed the generated amplicons. The reference sequences were NM_001845.4 (NP_001836.2) for COL4A1 and NM_001846.2 (NP_001837.2) for COL4A2. For the nucleotide numbering, the HVGS terms (www.hgvs.org) were applied with the nucleotide “A” of the ATG startcodon = c.1. The heterozygous variant c.2228G>T [NM_001845.4(COL4A1):c.2228G>T (p.Gly743Val)] was identified in exon 30 of the COL4A1 gene. The COL4A2 test was negative. The variant was found in IV-3 and IV-5 and not in asymptomatic relatives (III-4, IV-1, IV-4). The variant was confirmed by bidirectional fluorescence DNA sequencing (Sanger method).

### Bioinformatics Interpretation of Results

Interpretation of variant significance was done according to the American College of Medical Genetics and Genomics (ACMG) standards and guidelines (20). The p.Gly743Val variant is a conservative substitution that occurs in a position highly conserved across species (SIFT analysis: Deleterious–Score 0, median: 4.22, highly conserved nucleotide and amino acid, up to Tetraodon considering 11 species) and affects a crucial and abundant residue within the triple-helix-forming collagenous domain of the protein, which consist of long stretches of Gly-X-Y repeats. Combinations of the *in silico* tool MutationTaster® (21) and the Alamut® software (ALAMUT package, http://www.interactivebiosoftware.com, France) predicted the variant to be pathogenic as it likely alters the protein structure/function due to a detrimental effect on α1α1α2 heterotrimers formation and type IV collagen stability.

### Systemic Work-Up

After the COL4A1 mutation was found, systemic manifestations of COL4A1 mutations were investigated. No patient had cramps, cardiac symptoms, or abnormalities or Raynaud phenomenon. Systemic work-up including renal function, CK levels, urinary sediment test, and renal ultrasound proved unremarkable.

### Therapeutic Intervention

One patient (IV-3) was treated for spasticity and seizures with valproic acid. Lenses corrected for hypermetropia.

No ophthalmological surgery was planned on annual control for any member, but only “positive” lens correction prescribed.

## Discussion

We describe, here, the phenotype of a likely pathologic variant (p.Gly743Val) in exon 30 of the COL4A1 gene, responsible for an oculo-cerebral phenotype characterized by severe hypermetropia and highly penetrant porencephaly in absence of other systemic complications.

COL4A1 codes for extracellular matrix proteins that form heterotrimers that are major components of nearly all organ basal membranes. Clinically, COL4A1 mutations are responsible for different overlapping phenotypes including porencephaly (2–4), brain small vessel disease (2, 5–7) with or without ocular anomalies, HANAC (13) (hereditary angiopathy with nephropathy, aneurysms, and muscle cramps) syndrome, ophthalmological abnormalities (9–12), and non-syndromic autosomal dominant congenital cataracts (10). The COL4A1 gene has 52 exons and most of the pathogenic variants are distributed across exons 10 to 47 in the triple-helix domain. The pathogenic mechanisms of COL4A1 mutations are not fully elucidated and may vary according to the mutation type, the affected exon (mutations responsible for systemic HANAC syndrome cluster at exon 24 and 25), the position of the mutation within the triple-helix domain, and the mutation location. For instance, retinal arteriolar tortuosity relates to mutations in the amino-terminal one-third of the protein while mutations causing cataracts and ocular morphologic alterations are more likely to occur, closer to the carboxy terminus (22), like the variant we report.

We believe that the variant p.Gly743Val is likely pathogenic for several reasons. Firstly, it segregates within the family with the phenotype. Secondly, the p.Gly743Val variant is a missense mutation that shares features with other missense pathogenic mutations that occur in the COL4A1 gene exon 30: congenital porencephaly, epilepsy, and neuropsychological anomalies in p.Gly749Ser (23, 24), ophthalmologic defects and neuropsychological deficits in absence of systemic signs in variant p.Gly755Arg (25–27), and antenatal fetal intracerebral hemorrhage, ocular anomalies associated to cerebral leukoencephalopathy in variant p.Gly773Arg (12, 28, 29). Thirdly, bioinformatic tools and ACMG (20) classify p.Gly743Val as “likely pathogenic” due to the combination of the following criteria: (i) the p.Gly743Val variant is located in a mutational hotspot/or critical and well-established functional domain, (ii) the p.Gly743Val variant is absent from controls in the Exome Sequencing Project as reported by GeneDx (30), (iii) the p.Gly743Val variant is a gene that has a low rate of benign missense variation and in which missense variants are a common mechanism of disease, (iv) the variant p.Gly743Val has been previously reported, without phenotypic description in one other report [GeneDx Accession: SCV000531635.4 Submitted: (January 29, 2019)] and from one likely “pathogenic” [Undiagnosed Diseases Network, NIH Accession: SCV000926981.1 Submitted: (February 21, 2019)], and (v) which multiple lines of computational evidence support a deleterious effect on the gene product (see the Bioinfromatic Interpretation of Results).

This variant p.Gly743Val combines hypermetropia in all heterozygotic patients and highly penetrant antenatal porencephaly (associated with motor and intellectual deficits). Yet, as for all COL4A1 mutations, no specific treatment is currently available, and, due to the variable penetrance, adapted follow-up is challenging. For asymptomatic patients, cerebral and vessel imaging for aneurysm screening and ophthalmologic follow-up are indicated (2). Cesarean delivery for pregnancies with fetus at risk for a COL4A1-related disorder is recommended to prevent brain vascular injury attributable to birth trauma during delivery (6).

The strengths of our study are the extensive systemic work-up, the 5-year neurological follow-up, and the pluridisciplinary approach. Supporting children in their development to reduce handicaps and combining their follow-up with parent counseling could be considered as an ideal approach.

The limitations include the limited number of tested members (only two generations) due to a large family spread over Europe and not fully accessible. Yet, five siblings, showing mild phenotype even in the second generation support a Mendelian transmission with variable expressivity and no other mechanism. Another limitation is the systemic work-up based on described phenotypes and supposed affected organs. However, in rare pathologies with few cases, we may have missed undescribed or subclinical manifestations.

### Patient Perspective

III-3 was informed of the genetic diagnosis and is now regularly followed and screened for cataracts and brain aneurysms. IV-3 and IV-6 are closely followed by a neuropediatrician (VW). IV-3 goes to a normal school, but special schooling is required for IV-6. At the age of 12, IV-3 underwent cerebral palsy quality of life (CPQoL) questionnaires in which they expressed a satisfactory quality of life and a good relationship with other children. Genetic counseling will be proposed when IV-3 and IV-6 intend to start a family as there is a 50% risk of mutation transmission to the next generation and potential obstetrical complications.

## Conclusions

We described the phenotype associated to a likely pathogenic variant of the COL4A1 gene (c.2228G>T, p.Gly743Val) responsible for severe hypermetropia and familial porencephaly. This variant highlights that the COL4A1 mutation should be sought in cases of familial ophthalmologic pathologies associated with congenital porencephaly or early onset leukoencephalopathy.

## Ethics Statement

Written informed consent was obtained from the patient and the patient's parents for publication of this case report.

## Author Contributions

PS: wrote thi paper and performed the review of the literature under the supervision of GN. PS and NL: followed III-3 at the Erasme Neurology outpatients clinic. PV and VW followed the children at the Neuropediatrics clinic of the same hospital. All authors contributed to the article and approved the submitted version.

## Conflict of Interest

The authors declare that the research was conducted in the absence of any commercial or financial relationships that could be construed as a potential conflict of interest.
